# 40 Years of RAS—A Historic Overview

**DOI:** 10.3390/genes12050681

**Published:** 2021-05-01

**Authors:** Alberto Fernández-Medarde, Javier De Las Rivas, Eugenio Santos

**Affiliations:** Centro de Investigación del Cáncer-IBMCC (CSIC-USAL) and CIBERONC, Universidad de Salamanca, 37007 Salamanca, Spain; jrivas@usal.es (J.D.L.R.); esantos@usal.es (E.S.)

**Keywords:** RAS, milestones, GTPases, cancer, history

## Abstract

It has been over forty years since the isolation of the first human oncogene (HRAS), a crucial milestone in cancer research made possible through the combined efforts of a few selected research groups at the beginning of the 1980s. Those initial discoveries led to a quantitative leap in our understanding of cancer biology and set up the onset of the field of molecular oncology. The following four decades of RAS research have produced a huge pool of new knowledge about the RAS family of small GTPases, including how they regulate signaling pathways controlling many cellular physiological processes, or how oncogenic mutations trigger pathological conditions, including developmental syndromes or many cancer types. However, despite the extensive body of available basic knowledge, specific effective treatments for RAS-driven cancers are still lacking. Hopefully, recent advances involving the discovery of novel pockets on the RAS surface as well as highly specific small-molecule inhibitors able to block its interaction with effectors and/or activators may lead to the development of new, effective treatments for cancer. This review intends to provide a quick, summarized historical overview of the main milestones in RAS research spanning from the initial discovery of the viral RAS oncogenes in rodent tumors to the latest attempts at targeting RAS oncogenes in various human cancers.

## 1. The Discovery of RAS Genes: From Viruses to Humans and Beyond

### 1.1. The Retroviral RAS Oncogenes

The earliest studies leading to RAS discovery date from the late 1960s. Almost twenty years before the identification and isolation of the first human RAS oncogene, JJ Harvey isolated a transforming retrovirus from leukemic rats that was capable of producing sarcomas in infected rodents. The virus was named Harvey-MSV (Murine Sarcoma Virus) and shown to be the result of the recombination between retroviral genomes and a cellular rat gene (named Harvey-RAS, Ha-RAS, the name coming from for rat sarcoma) that was responsible for the transforming ability of this virus [1]. Shortly after, a different mouse retrovirus was isolated with the ability to induce erythroblastosis and sarcomas after several passages in newborn W/Fu rats [2], and subsequent work also demonstrated a recombinational mechanism of origin for this new transforming retrovirus that involved, in this case, a different (but related) cellular rat gene (designated Kirsten-RAS, Ki-RAS) [3]. The same lab also made a number of important contributions to the characterization of these genes, including the discovery of mammalian cellular homologs of the viral RAS oncogenes [4] and the demonstration that these oncogenes were different from the paradigm *src* viral oncogene and coded for small proteins (21 kDa) able to bind guanine nucleotides [5]. From 1981 onward, the name “RAS” was agreed for the viral transforming oncogenes of the Ha-MSV and Ki-MSV retroviruses, and the previously used p21-*src* denomination was also switched to p21-*RAS* [6].

### 1.2. The Human RAS Oncogenes

**Figure 1:**
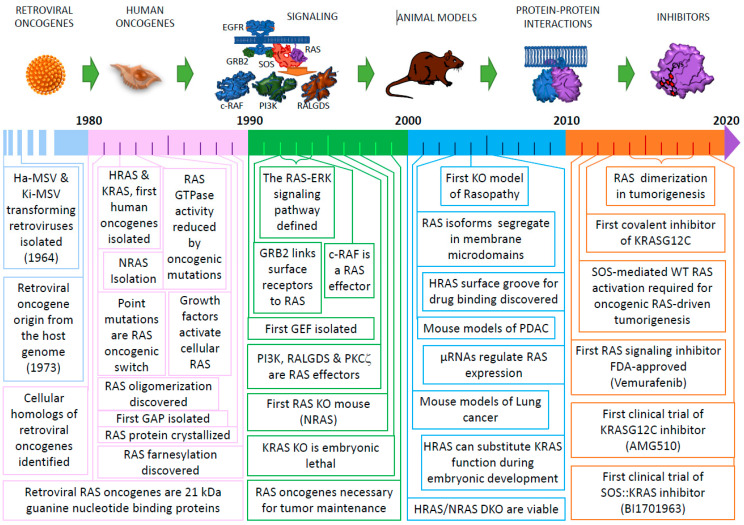
Milestones in RAS research. Summarized timeline of some key discoveries in RAS research over the last 40 years. We apologize for many key discoveries that could not be included here due to lack of space. The individual boxes point to the year of publication of some selected research advances that significantly contributed to the path leading to our current understanding of RAS structure, function and biology.

The greatest step forward in our understanding of the origin of human cancer and the role of the RAS GTPases in tumor development came at the beginning of the 1980s, with a flurry of independent reports produced by several different labs of the US east coast (Boston: MIT and Dana Farber. Long Island: Cold Spring Harbor Labs. Bethesda, Md: National Cancer Institute) (Figure 1). Working independently with DNA extracted from a human bladder carcinoma cell line (T24 or EJ), these laboratories used recently developed transfection procedures and hybridization assays using probes from viral oncogenes and repetitive human (*Alu*) sequences to identify and clone the first human oncogene (T24, now HRAS), which happened to be a human homolog of the previously known viral Harvey-RAS oncogene [7,8,9]. Soon after, similar experimental approaches using DNA isolated from various other cell lines and tumors led to the isolation of a much larger gene, designated KRAS, because it was shown to be the human homolog of the viral Ki-RAS oncogene [7]. Finally, a third member of the canonical human RAS gene family (designated N-RAS) was identified and isolated a few months later from a human neuroblastoma cell line and from other sarcoma cell lines, although a related viral oncogene did not exist in this case [10,11]. In parallel, various characterization studies quickly confirmed the individuality of the three human RAS oncogenes detected in transfection assays and mapped them to specific mouse and human chromosomal locations; in particular, the human HRAS, KRAS and NRAS genes are located, respectively, in chromosomes 11, 12 and 1 [12,13,14].

The most striking discovery concerning the human RAS oncogenes and their relevance for the mechanisms of carcinogenesis was the surprising observation (at that time) that all three human RAS oncogenes became oncogenically activated through the acquisition of a single point mutation affecting one of two main hot spots, located around codons 12 or 61 of their primary nucleotide sequences [15,16,17].

Following the seminal observations establishing single point mutations as the oncogenic switch for RAS genes, extensive screenings from many laboratories worldwide analyzing different tumor types and stages confirmed the specific presence of mutated RAS oncogenes in a large variety of specific hematological and solid tumors (particularly, lung, colon and pancreatic cancers) (early review in [18]). In this regard, two 1984 reports demonstrating the presence of a KRAS mutation in lung or ovarian carcinoma tissues, but not in normal tissue of the same patient [19,20], provided conclusive conceptual proof and a significant step forward in the scientific saga leading to the demonstration of a specific causal relationship between RAS oncogenic mutations and the development of specific tumors in patients.

From a mechanistic point of view, some reports were also relevant as they showed that simple RAS mutations are not enough for malignant transformation of primary fibroblasts. In particular, transfection studies using RAS oncogenes showed that fibroblast immortality is a prerequisite for transformation by the HRAS oncogene [21], and that tumorigenic conversion of primary embryo fibroblasts requires transfection of a separate, cooperating oncogene (MYC or Large T antigen) in addition to the RAS oncogene [22].

### 1.3. The RAS Gene Products

The discovery and isolation of RAS genes was immediately followed by the characterization of their transcriptional and translational products. In this regard, this was relevant as it led to, for example, the discovery of alternative splicing of the K-RAS transcripts in mice [23], a discovery which was also soon confirmed in humans [24].

The development of expression vectors and experimental procedures allowing the exogenous expression and the purification of large amounts of activated RAS proteins contributed greatly to their subsequent biochemical characterization. These early biochemical studies using purified RAS proteins produced the crucial discovery that RAS is a GTPase and that the oncogenic mutations produce a strong reduction of their intrinsic GTPase activity [25], revealing that oncogenically active RAS has GTP bound to its protein moiety and that its GTPase activity inactivates its growth-promoting effects. 

Another important milestone in RAS research was the discovery of post-translational modifications at the C-terminal region of RAS proteins which targeted these otherwise hydrophilic proteins to the plasma membrane [26,27]. Whereas initial biochemical analyses identified the presence of palmitate attached to the C-terminal, hypervariable region of RAS proteins [28], later studies inhibiting the mevalonate pathway identified covalently bound farnesyl isoprenoid residues as the critical hydrophobic molecules responsible for the anchorage of RAS proteins to the inner side of the plasma membrane (Figure 1) [29,30]. These discoveries provided the conceptual basis for the development, mostly during the 1990s, of multiple RAS farnesyl transferase inhibitors (FTIs) intended for use as potential, targeted therapeutic drugs against RAS-driven cancers (see early review [31]).

### 1.4. The Universal RAS Superfamily in Eukaryotes

**Figure 2:**
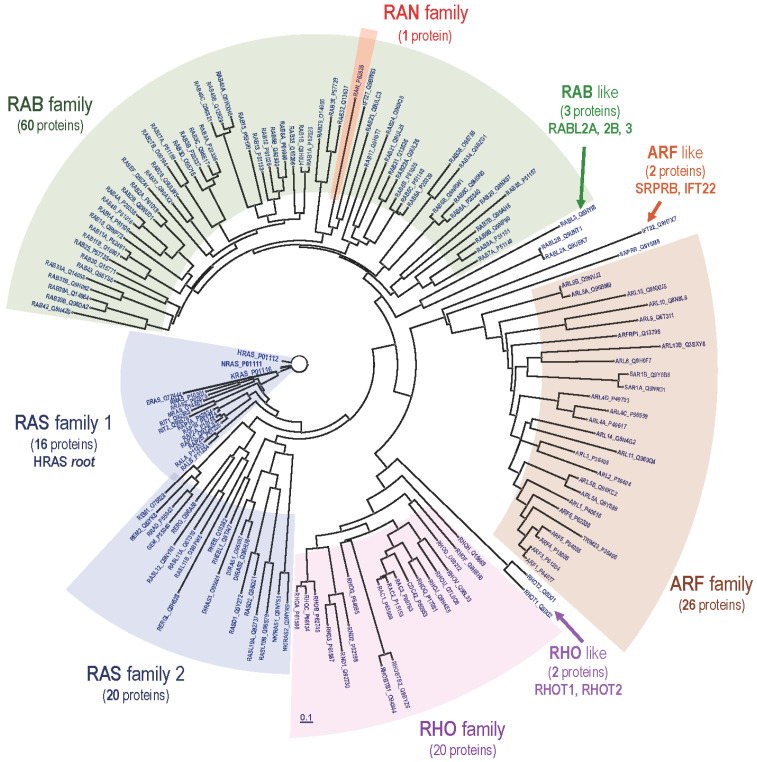
The RAS superfamily. Protein phylogenetic tree, including 150 human small GTPases corresponding to distinct, protein-coding gene loci (proteins annotated in the UniProt database). The HRAS sequence was used as the root for tree construction based on the distance matrix derived from the multiple alignment of the 150 amino-acid sequences using the neighbor joining method [33]. The tree includes 36 members of the RAS family (blue), 20 members of the RHO/RAC family (purple), 26 of the ARF family (brown), 60 of the RAB family (green) and 1 of the RAN family (red). Seven proteins that are frequently unclassified are identified here (pointed by arrows in the figure) as RHO-like (2), ARF-like (2) and RAB-like (3).

After the initial isolation and characterization of the human RAS oncogenes in the first half of the 1980s, the search for related genes and gene products using specific antibodies and nucleic acid probes gave rise, during later years, to the identification and characterization of a wide superfamily of related, monomeric small GTPases sharing structural and phylogenetic similarities with the original, H, N and KRAS oncogenes and acting as signaling molecular switches controlling the activation of many essential cellular functions. The three canonical HRAS, NRAS and KRAS oncogenes represent a very small part of the RAS subfamily, whose members act in control of processes of proliferation and differentiation. The members of the more distantly related RHO/RAC, ARF, or RAB subfamilies are known to participate in the regulation of processes of intracellular vesicle movement and cytoskeletal organization (reviewed in [32]). Figure 2 provides an updated, evolutionary tree of the proteins belonging to the various subfamilies composing the overall RAS superfamily, starting from the canonical HRAS, NRAS and KRAS oncoproteins that were originally discovered [33].

Analysis of the expression of RAS and RAS-related genes along the evolutionary scale soon demonstrated that the RAS GTPases are widely expressed in all eukaryotic cells and organisms. Thus, early screenings using antibodies generated against viral RAS proteins immediately showed the expression of related proteins in many different mammalian species including primates (humans, chimps, rhesus and capucinus monkeys (*Macaca mulatta,* *Cebus capucinus*)), rodents (hamsters, rats and several mouse species (*Mus musculus*, *M. caroli* and *M. cervicolor*)), as well as mink and horses [4]. Subsequent screenings also identified the presence of RAS genes and proteins in yeast (*Saccharomyces cerevisiae*, *Schyzosaccharomyces pombe*) [34,35], invertebrates (*Drosophila melanogaster*, *Caenorhabditis elegans*) [36,37] or lower vertebrates such as *Xenopus laevis* [34]. Interestingly, plants and microorganisms appear to lack the canonical RAS proteins, although they contain small GTPases controlling growth, proliferation or movement that have a closer resemblance to the members of the mammalian RHO/RAC or the RAB/ARF subfamilies (reviewed in [38]).

## 2. The Progress of RAS Biology and Signaling during the 20th Century

In the last quarter of the past century, multiple separate contributions from different labs around the word provided a multifaceted, multipronged path of research and discovery that eventually produced seminal experimental advances and conceptual milestones regarding the basics of RAS structure, function and biology (Figure 1). The following subsections summarize some of the conceptual advances produced during that period that helped setting the path of progress toward our current understanding of RAS biology and the regulation of the RAS signaling cycle.

### 2.1. Growth Factors and Their Receptors Activate Cellular RAS

The discovery and characterization of the human RAS oncogenes and the demonstration of their involvement in tumorigenesis set up the starting point for a race to uncover their role in different types of cancer and to find the signaling events that regulate, and are regulated by, these small GTPases. Between 1984 and 1987, most published reports on RAS function focused on analyzing its causal role in different types of human cancer, but very few described mechanistic aspects of RAS regulation of cellular signaling. Highly relevant in this regard were the initial discoveries that uncovered the participation of RAS proteins in signal transmission from growth factor receptors showing that EGF stimulates guanine nucleotide binding to RAS proteins [39] but that EGFR stimulation is not necessary for oncogenic RAS activation [40]. We may also mention other early reports showing that oncogenic RAS is also an essential intermediate of the action of insulin, inducing meiotic maturation of *Xenopus* oocytes and its associated phosphorylation events [41,42], or characterizing the involvement of RAS proteins in the activation of inositol phosphate signaling pathways and related second messengers [43,44].

### 2.2. RAS Activation. The Guanine Nucleotide Exchange Factors (GEFs)

A major advance in RAS-signaling biology derived from the discovery in 1987 of a novel yeast gene whose mutations triggered similar effects to RAS mutations in Saccharomyces cerevisiae. The gene was named CDC25 and was proposed to be a cellular activator of RAS GTPases by regulating nucleotide binding to RAS proteins [45]. A few years later, this initial study led to the discovery of guanine nucleotide exchange factors (GEFs) in mammalian cells [46] and the isolation of the first RAS GEF (CDC25Mm, now known as RASGRF1) [47]. In the following years, the trinity of canonical RASGEF families was completed with the isolation and characterization first of SOS in Drosophila and mammals [48,49], and then of RASGRP [50]. Other non-canonical GEFs have been described more recently, including GRASP1 (isolated in the CNS) [51], Very-Kind [52], RGR (an oncogene with RAS GEF activity) [53], and even PLD2 (which, in addition to its phospholipase activity, has been reported to have GEF activity on RAS) [54]. 

The most recent studies have demonstrated that the SOS GEFs are the most widely expressed and functionally relevant RAS activators in Metazoa, having a critical relevant role not only in individual cells but also in the control of organismal viability and homeostasis (rev. in [55]). In contrast, the RASGRFs exert most of their activating RAS functions in the central nervous system (rev. in [56]) whereas the RASGRPs are mostly involved in RAS activation in the hematopoietic system, regulating lymphocyte, mastocyte and macrophage maturation and function (rev. in [57]).

### 2.3. RAS Inactivation. The GTPase Activating Proteins (GAPs)

A few months after the discovery of CDC25, another important finding further clarified how the cycle of RAS activation/deactivation is regulated. Using extracts from *Xenopus* oocytes, as well as from mouse and human cells, a protein was identified that was capable of multiplying by many times the intrinsic GTP hydrolytic activity of cellular RAS proteins. It was also observed that the oncogenic mutations rendered RAS proteins insensitive to the effects of this factor, which was named “GTPase Activating Protein” (GAP) [58]. Shortly after came the demonstration of the physical interaction between this GAP and RAS at a region that had been previously hypothesized to be the effector domain, thus suggesting the consideration of GAP proteins as possible RAS effectors [59]. Parallel studies in other labs led to the purification, from cow’s brain, of the first mammalian RASGAP, a 125KDa protein that enhanced RAS GTPase activity by almost 100-fold and is now known as p120RASGAP or RASA1 [60]. Fourteen other mammalian RAS GAPs have been discovered ever since, including neurofibromin 1 (NF1, the protein responsible for neurofibromatosis type 1, which is also a RAS GAP closely related to the Saccharomyces IRA proteins) [61], the GAP1 family (GAP1m/RASA2, GAPIP4BP/RASA3, CAPRI/RASA4 and RASAL1) and the SynGAP family (SynGAP, DAB2ip, RASAL2 and RASAL3) (rev. in [62]).

### 2.4. The Link Between Surface Receptors and RAS Activation. The GRB2 Adaptor Proteins

By the end of the 1980s it was already clearly established that RAS proteins are located at the plasma membrane, where they receive and transmit signals from surface growth factor receptors, but the intermediate link between the surface receptors and RAS activation was still missing. The first insight in this regard came with the discovery of GRB2, an adaptor protein that was shown to bind to phosphorylated tyrosine residues of the intracellular domain of transmembrane receptors that had been activated by agonist binding [63]. The following step in this research was the demonstration that GRB2 was able to establish functional interactions with RASGEFs, triggering the increase in GEF exchange activity on RAS proteins at the plasma membrane [64]. In particular, this interaction was demonstrated almost at the same time for mouse and human SOS proteins [65,66]. All these discoveries eventually led to the design of a signaling scheme in which growth factor-dependent receptor dimerization and autophosphorylation led to recruitment of GRB2 and associated SOS GEF proteins to the plasma membrane, where they could find and activate their RAS targets to trigger intracellular signaling pathways [67]. This way, for the first time in 1994, we were able to complete a RAS pathway to the nucleus involving a continuous, linear sequence of interconnected signaling elements capable of internalizing extracellular signals and triggering activation of internal cytoplasmic MAP kinases, with RAS proteins acting as essential intermediates of the process.

### 2.5. RAS Functional Domains and Three-Dimensional Structure

Early comprehensive mutagenesis studies carried out in different laboratories allowed an initial identification of “essential” and “dispensable” functional domains located along the primary amino acid sequence of RAS proteins. For example, since mutations within a region stretching from position 32 to 40 greatly reduced the biological activity of HRAS proteins, this region was postulated as an essential domain responsible for the interaction of RAS with its effectors [68]. Later on, similar mutagenic approaches led to the identification of the regions responsible for RAS-GAP interaction [69] or for guanine nucleotide binding [70]. 

The first crystallographic structure of a RAS protein (purified, non-mutated HRAS lacking the last 18 residues) was available in 1988 and identified four α-helixes and six β-sheets connected by nine loops [71]. Analysis of this RAS crystal structure immediately showed basic structural similarities with other nucleotide-binding GTPases and enabled the process of ascertaining the spatial location and interactions of the different functional domains previously identified by mutagenesis; for example, the previously identified effector domain [68] was shown to be located in an exposed area of the protein [71]. Many subsequent studies have later completed different details of the 3D structure of RAS proteins. Among these, we may cite the crystallization of RAS protein bound to a GTP analogue that allowed to precisely define the amino acids responsible for GTP binding [72]. Later mutagenesis studies uncovered the relevance of the switch-I and switch-II in the interaction of RAS proteins with their activating GEFs [73,74]. Furthermore, work on yeast CDC25 identified the residues responsible for this interaction [75,76], and similar results were obtained with the analysis of mammalian SOS [77] and RASGRF [78]. Definitive, final confirmatory evidence for these proposed interactions was provided by the analysis of the crystal structure of a complex containing RAS combined with the catalytic region of SOS [79].

### 2.6. RAS Downstream Effectors and Signaling Pathways

By 1993, the backbone of the upstream signaling processes leading to RAS activation had already been described. However, the signaling events elicited after RAS activation were not yet completely understood. For example, we mentioned above that the first protein defined as a RAS effector was p120RASGAP [59], but it was unclear how a protein implicated in RAS inactivation could also act as an effector [80]. Indeed, we know nowadays that p120RASGAP can act as a RAS effector modulating several signaling pathways, including those controlled by RHO, Aurora kinase or AKT [81]. In any case, the discovery of the first “exclusive” effector of RAS was delayed until 1993, when it was shown that RAF-1 (c-RAF), the cellular version of the v-RAF oncogene, was able to bind to RAS. RAF-1/RAS interaction was only possible when RAS was bound to GTP and this led to subsequent activation of the MAPK cascade [42,82]. Once it became public knowledge that RAS interaction with its RAF effector was GTP-dependent, new effectors were soon discovered including PI3K [83], RALGDS [84], PKCζ [85] and so on (early rev. in [86]).

After the discovery of RAS effectors, the key question to be answered was how downstream signaling managed to translate the activation of each effector into specific elicited cellular responses. Regarding RAF effectors, prior reports showing MEK activation by c-RAF [87] and activation of ERK by MEK [88], provided the complementary evidence supporting the notion that RAS was able to activate the RAF/MEK/ERK MAPK cascade [82]. Soon after, other reports showed that MAPK is indispensable for AP-1 activation and fibroblast proliferation, and that introduction of constitutively active MAPK induces the FOS promoter and cell proliferation in the absence of growth factors [89,90]. Taken together, these observations showed for the first time that RAS stimulation of the RAF/MEK/ERK cascade leads to cell proliferation through the activation of transcription factors, thus transducing external signals that reach the cell surface to internal changes of gene expression in the nucleus. Many important aspects regarding PI3K as a RAS effector were also described in the 1990s, including the PI3K-mediated activation of AKT/PKB [91] and p70S6K [92], as well as its role in the regulation of protein synthesis and glucose transport and metabolism by insulin [93,94,95]. Similar experimental approaches have also provided the mechanistic explanations on how activated RAS proteins are able to use other effectors as mediators of a wide range of intracellular physiological or pathological responses including cancer (see early review [96]).

## 3. Further Advances on RAS Signaling during the 21st Century

The following subsections describe several additional advances on RAS biology that have been produced in more recent years, thus leading to an improved understanding of RAS regulation and function in the plasma membrane and other intracellular domains.

### 3.1. RAS Regulation Through Covalent Modifications

In addition to the early identification of palmitoyl and farnesyl binding to the C-terminal region, an increasing number of later reports have also shown that modulation of RAS activity is far more complicated and may rely also on many other covalent modifications. For example, whereas phosphorylation of RAS by PKC was discovered long ago [97], more recent reports have shown that PKC phosphorylation of KRAS in S181 regulates its location and signaling activity [98] and is necessary for KRAS-induced tumorigenesis [99]. 

Consistent with functional differences among the isoforms, it was also shown that HRAS or NRAS, but not KRAS, can be mono- or di-ubiquitinated, and this modification targets these two isoforms to endosomes [100]. Furthermore, RAS mono-ubiquitination not only induces a change of location but also decreases the sensitivity of RAS to GAP inactivation [101]. The differential phosphorylation and ubiquitination of RAS isoforms, together with the finding of the acylation/deacylation cycle of HRAS and NRAS [102], may account, at least in part, for specific physiological and pathological roles played by these two isoforms in various biological contexts. HRAS polyubiquitination by β-TrCP E3 ligase in association with previous threonine 144/148 phosphorylation by GSK3β has also been proposed as a mechanism to control HRAS stability. HRAS degradation by the proteasome was blocked by the WNT/β-catenin pathway linking WNT signaling and sustained RAS activation in cancer [103]. 

RAS glutathiolation in cysteine 118 has also been described as a posttranslational modification affecting RAS function that may provide a functional link between RAS proteins and cellular oxidative stress [104]. RAS proteins can also be nitrosylated, resulting in increased cell proliferation that is important for neurogenesis after seizure injury [105,106]. Finally, RAS can be also acetylated in lysine 104, a modification reported to negatively regulate RAS signaling by blocking RAS interaction with its GEFs, thus favoring the GDP-bound state [107].

### 3.2. RAS Inter-Molecular Interactions

Besides the intramolecular, covalent modifications of the RAS proteins, other regulatory mechanisms involving inter-molecular interactions have also been discovered in the last two decades. Early in RAS history, it was reported that RAS proteins are able to form homo-oligomers in vivo [108], but this discovery about RAS high-level structural organization received little attention for more than two decades. At the beginning of this century, a separate report described the existence of clusters of HRAS and KRAS in membrane microdomains that segregated each isoform [109], and the existence of these microdomains was later demonstrated for NRAS also [110]. Indeed, clustering of RAS proteins has been shown to be important for downstream signaling, and mutations blocking RAS association in microdomains have been shown to inhibit cell transformation by KRAS and HRAS [111,112]. Finally, more recent reports using physicochemical and imaging techniques on living cells [113,114] have confirmed that RAS GTPases can associate to form protein dimers in the cells that may be functionally relevant. Indeed, it could be speculated that the existence of dimers of MEK, ERK and RAS may be mechanistically relevant for the process of signal transduction *via* the RAS-ERK pathway [115]. Importantly, recent reports have shown that RAS dimerization lead to a better activation of ERK [114], and that oncogenic mutations stabilize dimerization of KRAS, drive tumorigenesis and may also account for tumor resistance to MEK inhibitors [116,117].

### 3.3. Positive Feedback Loops of RAS Activation

Another very relevant finding regarding the regulatory mechanisms of RAS activation was the discovery of an allosteric binding site for RAS in SOS1, showing that the specific binding of an activated RAS.GTP molecule to SOS1, in a region independent of the catalytic region responsible for nucleotide exchange, results in a significant increase in the overall exchange activity on other RAS molecules through the establishment of a positive feedback loop ensuring that many more inactive RAS molecules are then processively activated by the same allosterically activated SOS molecule [118]. Mutations in the SOS allosteric region are known to have profound functional consequences on RAS downstream signaling in different physiological and pathological contexts [55]. Importantly, in this regard, the allosteric activation of SOS1 GEF activity by WT RAS.GTP ultimately explains the seminal and somewhat surprising (at the time) demonstration of the critical requirement of the contribution of activated non-mutated WT RAS for the development of oncogenic RAS-driven tumors [119,120].

### 3.4. RAS Signaling from Endomembranes

The capacity to signal from endomembranes is another relevant concept added to our understanding of RAS biology and function in the last 20 years. Although the location of RAS proteins in the endoplasmic reticle (ER) and Golgi was known very early, it was thought to be merely an intermediate step during protein maturation and transport to the plasma membrane. This notion was revoked in 2002 by experiments using RAF1 RBD probes linked to GFP to show that a portion of RAS found in ER and Golgi is bound to GTP and able to interact with downstream effectors [121]. Therefore, RAS activation on different subcellular locations may lead to different levels of activation of effector pathways, suggesting a complex network of signaling efficacies and effects depending on the levels of each RAS isoform and their location [122,123].

### 3.5. Regulation of RAS Expression by Micro RNAs

Another relevant conceptual advance regarding RAS regulation and function was the discovery of Let7, a microRNA capable of controlling RAS expression in *C. elegans* [124]. Let7 is only the first of many other related microRNAs that have been later identified in mammalian cells with the ability to induce RAS mRNA degradation and downregulate RAS expression. Since these microRNAs are frequently downregulated in cancer, reversion of this alteration might be considered a new potential therapeutic option (rev. in [125]).

## 4. RAS in Physiology and Pathology

### 4.1. Central Role of RAS Signaling in Physiological Cellular Processes

The crucial role of RAS genes and proteins regarding the control of normal eukaryotic cell proliferation was demonstrated early on by means of experiments showing that the microinjection of neutralizing RAS antibodies inhibited the initiation of S-phase in NIH 3T3 fibroblasts cells, indicating that RAS was essential for G1-S progression in the cell cycle [126]. 

Besides controlling cellular proliferation and progression of the cell cycle, RAS proteins have also been found to regulate many other normal relevant cellular processes, including differentiation. For instance, viral RAS oncogenes were shown to alter differentiation of cultured epidermal cells [127], and human RAS oncogenes have also been shown to induce neuronal differentiation of pheochromocytoma PC12 cells, or differentiation of 3T3L1 fibroblasts into adipocytes [128,129]. 

Many other physiological cellular processes are also regulated by RAS proteins. For example, RAS proteins have been shown to induce expression of the autocrine motility factor, a cytokine responsible for cell migration [130] and to control cell migration mostly through its regulation of the RHO/RAC family GTPases [131,132]. Furthermore, RAS can also control intracellular vesicle trafficking through regulation of RAL GTPases [84].

The critical relevance of the RAS GTPases in the control of normal cellular functions is further underscored by the many different deleterious effects that may appear in individual cells and living organisms as a consequence of the alterations of normal RAS signaling and functions caused by the *gain-of-function* (hyperactivating) mutations that may occur in canonical RAS family members or other components of RAS signaling pathways. In fact, RAS signaling alterations constitute a common cause of cancer (somatic oncogenic mutations) but may also trigger a wide variety of other human illnesses, either as causal mutations or as symptom-related changes, such as the developmental defects caused by inherited *gain-of-function* mutations of various members of RAS signaling pathways.

### 4.2. Aberrant RAS Signaling in Cancer

The demonstration of the activation of the MAPK cascade by RAS, together with previous findings showing that oncogenic RAS mutants trigger increased MAPK phosphorylation and activity independently of receptor activation [133], indicated that mutant RAS-mediated, constitutive activation of the RAF/MEK/ERK kinase cascade leads to uncontrolled cell proliferation and eventual tumor development. This concept was later reinforced by detection of oncogenic mutations in various other members of the RAS-ERK pathway. As supporting evidence, we may mention the transforming activity of the viral v-RAF oncogene (a truncated version of c-RAF) [134] or the frequent detection of BRAF mutations in malignant melanomas, thyroid cancer or other malignancies at a lower rate [135]. MEK and ERK mutations are rare in cancer, but their contributing role in signal transduction from upstream oncogenes has been well established [136,137] 

Alterations in other branches of RAS downstream signaling pathways have also been linked to cancer. In particular, aberrant PI3K signaling is frequently seen in human malignancies as shown by the detection of oncogenic PIK3CA mutations in colon, brain and other cancer types, including breast and endometrial tumors [138]. Overexpression of this locus is also a common trait in cancers of the lung, esophagus, cervix and especially ovary, with almost 50% of tumors showing high PIK3CA levels (COSMIC database). In addition to oncogenic mutations, PTEN, the most important negative regulator of the PI3K pathway, also suffers *loss-of-function* in many human malignancies. In fact, the role of PTEN as a tumor suppressor was known before its role as an inhibitor of the PI3K pathway [139,140]. 

Another important concept, gleaned from studies on RAS signaling in cancer, is the notion of mutual exclusivity among the potential oncogenic mutations that may occur in different components of a shared linear signaling pathway. Indeed, the experimental observation has shown that the co-occurrence of oncogenic mutations in components of the RAS/RAF/MEK/ERK signaling pathway is practically non-existent, probably due to the fact that such co-expression leads to oncogene-induced senescence. This was first observed in human melanoma, where BRAF and NRAS mutations are more frequent but never concur in the same cancer [141], and is also true for KRAS and BRAF alterations in lung cancer [142]. In fact, overactivation of this signaling pathway leads to senescence or apoptosis [143]. On the other hand, mutations in other downstream signaling pathways, such as the PI3K pathway, are not exclusive with RAS mutations [144].

All RAS downstream effector signaling pathways have been associated with tumorigenesis. However, because of their crucial roles in regulation of proliferation, growth, cell migration, metabolism and apoptosis, the RAS-RAF/MEK/ERK and the RAS-PI3K/AKT signaling axes are believed to be most relevant for cancer development and, therefore, most therapeutic efforts so far have been focused on these two particular pathways [145].

### 4.3. Defective RAS Signaling in Developmental Syndromes

The bulk of the experimental data accumulated during the last decades of the 20th century established the causal role of somatic RAS mutations in the development of a variety of sporadic human tumors appearing during adult life. Conversely, more recent research from the first decade of the 21st century produced the somewhat surprising discovery of the occurrence of inherited, germline RAS mutations in association with a number of distinct hereditary familial developmental syndromes now collectively known as RASopathies or cardio-facio-cutaneous-syndromes [146]. Mutations in RAS genes or many other upstream or downstream components of the RAS signaling pathway have been identified as the cause of the defective cellular signaling that is responsible for the appearance of these pathological conditions of development [147]. The first suspicion of a causal link between these kinds of hereditary syndromes and defective RAS signaling arose when the loss of neurofibromin 1 (NF1), known to be a GAP for RAS GTPases [61] was found in association with the development of neurofibromatosis 1, one of the most common RASopathies. Regardless, the starting milestone in this field came with the discovery that germline HRAS mutations are responsible for development of Costello syndrome. Of particular relevance was the observation that the range of HRAS mutations found in Costello patients had lower potency than those found in sporadic cancers, with worse prognosis and higher tumor predisposition rates in those mutations having stronger RAS activation capacity [146]. Soon after, other laboratories showed that *gain-of-function* germline mutations in RAS-GEFs, such as SOS1, are responsible for another related defect: designated Noonan syndrome. In this case, the increased GEF activity of a mutated SOS1 would be responsible for the hyperactivation of the signals coming from the RAS/ERK pathway [148]. Various other reports have also documented the widespread association of mutations in different components of RAS/RAF/MEK/ERK signaling pathways with the development of a variety of distinct, related developmental syndromes (see rev. [149]).

Finally, other recent reports have also found evidence supporting the implication of altered RAS signaling in non-tumoral illnesses including diabetes, Alzheimer’s, intellectual disability or schizophrenia, among others [150,151,152].

## 5. Animal Models for the Analysis of RAS Function

### 5.1. Transgenic Mice

Genetically modified murine strains are highly instrumental in analyzing the physiological and pathological roles played by specific genes/proteins in different biological contexts. This was quickly made evident for RAS oncogenes when the first transgenic mice bearing an HRAS^G12V^ mutation were shown to harbor teratocarcinomas developed early during embryonic development, thus pointing to the lethality of germline RAS mutations [153]. Afterwards, many different transgenic strains were generated that have helped to study and characterize the functional association between mutations in different RAS isoforms and the development of particular types of cancer. Transgenic mice have also been instrumental in analyzing chemical carcinogenesis [154] and for preclinical studies of anticancer drugs [155]. 

Early seminal reports analyzing mouse strains harboring HRAS^G12V^ and KRAS^G12D^ transgenes provided initial, direct evidence of the role of RAS oncogenes in cancer initiation and development by demonstrating the critical requirement of RAS oncogenes for tumor maintenance and the regression of tumors after RAS transgene expression was silenced, even in the absence of tumor suppressors such as INK4a or p53 [156,157]. Separate reports soon also characterized the ability of a KRAS^G12D^ transgene to develop mouse lung tumors resembling human NSCLC and also pancreatic tumors [158]. Other relevant reports showed that HRAS and KRAS transgenic mice developed different types of cancer, even when expressed under the same regulatory sequences, showing that small differences in oncogenic cell signaling may account for very different outcomes [159].

### 5.2. Knockout Mice

Whereas the study of transgenic mice produced significant, initial insights regarding RAS tumor biology, the generation and analysis of knockout mouse models has been crucial for understanding the normal physiological role of RAS proteins. The first report of a RAS knockout described that ablation of NRAS expression by gene targeting did not produce any gross phenotype, indicating the dispensability of NRAS for mouse development, fertility and growth [160]. In contrast, two later reports demonstrated the critical requirement of KRAS for mouse development, describing the embryonic lethality (between days 12 and 14) of KRAS-ablated embryos, which showed significant cardiovascular and liver defects, among others [161,162]. Finally, the analysis of HRAS-ablated mice showed that they were also perfectly viable [163] and, consistently, adult double HRAS^−/−^/NRAS^−/−^ knockout mice were also viable and presented no obvious phenotypic alterations [164].

### 5.3. Functional Specificity/Redundancy of RAS Isoforms

Despite the strong evidence for the functional specificity of the H, N and K RAS genes, detailed analysis of the biology of different RAS KO strains point also to at least some partial functional overlapping among these RAS isoforms in vivo. For example, KRAS^+/−^/NRAS^−/−^ die either during embryonic development or soon after birth, showing that the viability of adult NRAS KO mice is dependent upon a fully normal dose of KRAS expression [160]. Similarly, less than expected mendelian ratios of HRAS^−/−^/NRAS^−/−^ double KO mice were always obtained in crosses between HRAS KO and NRAS KO mice, and the resulting adult DKO showed low fertility rates, suggesting overlapping functionalities between HRAS or NRAS regarding embryonic development, postnatal growth and fertility [161]. Consistently, newborn HRAS^−/−^/NRAS^−/−^ DKO pups die between P0 and P2 due to defects in lung maturation, demonstrating functional redundancy for HRAS and NRAS in the last stages of lung development, where KRAS could not replace their function [162]. It was also shown that expression of HRAS under the control of the KRAS promoter in KRAS KO embryos allowed the birth of viable offspring at expected mendelian rates, although these adult mice suffered from high blood pressure and cardiomyopathy, suggesting that HRAS can replace KRAS function during embryonic development and that KRAS may play critical roles for normal cardiovascular physiology [163]. 

Other reports support the existence of overlapping functions between HRAS and KRAS. In particular, it was shown that double HRAS^−/−^/KRAS^−/−^ DKO embryos die in utero at earlier times than single KRAS KO embryos, suggesting overlapping functionalities of HRAS and KRAS, at least between E9.5 and E11.5. Consistently, the same report showed that expression of a human HRAS transgene under its natural promoter was able to rescue the lethality of KRAS KO mutant embryos, even in the context of a triple HRAS/NRAS/KRAS knockout [164]. These observations suggested that the normal dependence on K-RAS is based on promoter strength or specific spatiotemporal expression rather than the particular signaling characteristics of the KRAS protein. Somewhat in contrast to this notion, other studies using transgenic mice harboring KRAS or HRAS oncogenic mutations suggested that specific signals produced by the different RAS oncogenes may account for the different types of tumors developed by the mice harboring those transgenes [159]. Altogether, these analyses suggest that the non-mutated RAS isoforms show higher level of functional overlapping (acting in normal/physiological processes) than their corresponding oncogenic versions, which appear to be more functionally specific regarding the development of distinct types of tumor [165].

### 5.4. Mouse Models of RAS-Driven Cancer

The early transgenic mouse models carrying RAS mutations clearly demonstrated the causal relation between RAS oncogenes and tumor development but were not optimally designed to analyze the fine mechanistic and biological details related to in vivo tumor development of human cancers under different biological contexts. To overcome these limitations, new targeting strategies, allowing spatiotemporal induction of oncogenic mutations expressed under their native RAS gene promoters or breeding with genetically modified mice carrying other relevant mutations, have been later developed to allow the generation of rodent cancer models better recapitulating the behavior of human cancers.

A seminal report in this regard used a doxycycline-inducible HRAS^V12G^ transgenic mouse model that was crossed with a KO mouse strain lacking the tumor suppressor INK4a to generate offspring that developed malignant melanoma, but the skin lesions disappeared when doxycycline treatment was stopped, showing a critical role of RAS for tumor maintenance [156].

This notion was later confirmed and extended in a model of lung cancer where transgenic mice expressing murine KRAS4b^G12D^ in type II pneumocytes under the control of doxycycline developed lung tumors upon doxycycline treatment (indicating that KRAS mutations are necessary for tumor initiation), but the tumors regressed upon doxycycline removal (showing that KRAS is crucial for tumor maintenance). Furthermore, the tumors were more aggressive when the KRAS4b^G12D^ mice were crossed with strains lacking tumor suppressors such as p53 or INK4A/ARF, but even maintenance of these tumors was dependent on a sustained KRAS4b^G12D^ expression [157].

The role of KRAS in tumor initiation was confirmed using more physiological lung cancer models, in which adenovirus carrying the Cre recombinase was instilled into the nose of mice to induce expression of KRAS^G12D^ only in the lungs. In addition, these models allowed also the analysis of the stages of lung tumor progression (K-RASLA1 and K-RAS^LA2^ mice [166], K-RAS^+/LSLG12Vgeo^ mice [167]). Consistently, a separate mouse model of lung cancer designed to achieve expression of the oncogenic KRAS^G12D^ allele after a somatic event of homologous recombination developed mostly lung adenocarcinomas, which were more aggressive after partial or total elimination of p53 [168].

RAS mutation frequency is highest in pancreatic ductal adenocarcinomas (PDAC). Using a KRAS transgene expressed under control of pancreas-specific promoters (PDX-1 or P48), both PDX-1^Cre^;LSL-KRAS^G12D^ and P48^+/Cre^;LSL-KRAS^G12D^ mice developed pancreatic intraepithelial neoplasias (PanIN) which evolved to PDAC and metastatic disease, a tumor evolution resembling human PDAC, but the tumor incidence in these mice was very low [169]. In a later mouse model of PDAC harboring a specific genotypic combination (K-RAS^+/LSLG12Vgeo^;Elas-tTA;TetO-Cre), the oncogenic KRAS mutation was induced upon doxycycline treatment. These mice developed PDAC with a very similar pattern to the Cre;LSL-KRAS^G12D^ and P48+/Cre;LSL-KRAS^G12D^ mice, and the tumors were more aggressive in the absence of the p53 or INK4a/ARF tumor suppressors. Interestingly, pancreatic acinar cells of adult mice were resistant to oncogenic transformation by the KRAS^G12D^ oncogene, even in the absence of p53 [170,171].

A mouse model of melanoma was generated by introducing an NRAS transgenic allele under the control of the tyrosinase promoter in a INK4a^−/−^ KO mouse strain, showing that genetic lesions found in human melanoma are able to induce malignant melanoma in mice [172].

A murine model of KRAS induced colorectal carcinoma (CRC) was generated crossing the KRAS^L1^ mice with another mouse strain carrying the Cre recombinase under control of the Villin promoter. These mice developed colon cancer only in the proximal colon, showing that the molecular events leading to CRC are different depending on the region of the colon [173]. A more physiological model of CRC has been recently generated, in which mice bearing a truncated adenomatous polyposis coli (APC) floxed allele were crossed with mice carrying a heterozygous KRAS^G12D^ floxed allele and with a tamoxifen-inducible Cre recombinase model. The resulting mice developed CRC in a tamoxifen dose-dependent manner, with tumors being indistinguishable from human CRC [174].

Mouse models of cancer have been highly instrumental to better understand tumor progression in humans and are also useful tools for preclinical studies analyzing the therapeutic usefulness of drugs previously developed and tested in vitro or on cell cultures. The development of complex strategies producing mouse models growing tumors that closely resemble human cancer has allowed the generation of several mouse strains usable as preclinical models in drug development and have given new insights to the role of RAS GTPases in human malignancies (for a review of KRAS models of lung and pancreatic cancer, see [175]).

### 5.5. Animal Models of Human RASopathies

There are also specific animal models allowing the study of mutations in the RAS pathway that are involved in RASopathies. The first such model involved genetic targeting of the NF1 locus to generate mice that developed symptoms very similar to human neurofibromatosis [176]. Corresponding with the establishment of the link between defective RAS signaling and inherited developmental syndromes at the beginning of this century, the next relevant mouse model involved the knock-in of a G61D mutation in the Ptpn11 gene to generate a mouse strain recapitulating Noonan syndrome symptoms [177]. Likewise, the first mouse model for Costello Syndrome carried a G12V mutation in the HRAS gene and also recapitulated most of the symptoms observed in human patients [178]. Later reports have also described the generation and characterization of mouse models of different cardio-facio-cutaneous syndromes, aiming at understanding how germline mutations in components of RAS signaling pathways may lead to facial and skin defects, cardiomyopathy and various other symptoms observed in human patients (rev. in [179]).

## 6. Therapeutic Approaches to RAS-Driven Cancer

### 6.1. Blocking RAS Location at the Plasma Membrane

The initial search for therapeutic approaches targeting oncogenic RAS in cancer was for the most part a sad history of trial and failure. Early crystallographic studies suggested that finding blockers of RAS action would be a tough task with improbable success because the analysis of RAS structure did not show surface pockets where small molecule inhibitors could bind and block oncogenic signals [180]. Due to the unyielding nature of the RAS proteins, the initial focus of anti-RAS cancer therapeutics was placed on targeting RAS plasma membrane localization, mediated through the binding of isoprenoid residues to its C terminal region. For this purpose, a wide range of farnesyl transferases inhibitors (FTIs) were developed in different laboratories with the goal of blocking RAS signaling from the plasma membrane [31]. In short, despite initial success blocking RAS farnesylation in tissue culture and preclinical models, FTIs failed completely in clinical trials (rev. in [181]), probably due to the fact that KRAS and NRAS can also be geranyl-geranylated (another form of prenylation used by cellular proteins to gain access to the membrane) [182]. The next logical step was, therefore, to design dual prenylation inhibitors, but dose-dependent toxicity has prevented these molecules from reaching clinical use so far [183]. Despite these uninspiring results, new attention has lately been put on the use of refined FTIs for cancer treatment, and as on April 2021, several clinical trials are being carried out; for example, testing Tipifarnib on tumors harboring HRAS mutations (see https://www.clinicaltrials.gov accessed on 5 April 2021, for more details). 

Alternative strategies to delocalize RAS oncoproteins have also been developed recently. One of these involves the use of a novel inhibitor designed to disrupt the recently discovered interaction between KRAS and PDEδ, which is needed for KRAS placement in the inner side plasma membrane. This inhibitor, named Deltarasin was reported to prevent KRAS location to the plasma membrane and block pancreatic cancer cell proliferation [184]. A different approach takes advantage of RAS prenylation by binding covalent inhibitors to RAS through the action of the farnesyl transferase. This prevents the action of geranyl-geranyl transferases and mislocalizes KRAS in SW-620 colon cancer cells [185].

### 6.2. Inhibiting RAS Downstream Signaling

The lack of effectiveness of FTIs in clinical trials, together with the absence of discernible pockets on the RAS surface allowing direct binding of inhibitor, led to the assumption that RAS could be “undruggable” and for many years the search for drugs against RAS-driven tumors focused on the inhibition of downstream signaling pathways [186]. Most downstream RAS effectors are protein kinases, enzymes for which inhibitors are easily developed, as proven by the vast availability of inhibitors of the MAPK cascade. So far, among the inhibitors developed against RAF, MEK and ERK1/2, the RAF inhibitors hold the most promise for cancer treatment. In addition to its role as an effector of oncogenic RAS, RAF is also frequently mutated in cancer. Unfortunately, the first generation of RAF inhibitors showed high rates of drug resistance and subsequent analysis of the underlying resistance mechanisms uncovered a paradoxical hyperactivation of ERK, either through increased activation of WT RAF dimers in BRAF mutant cancers, or by transactivation of the drug-free RAF molecule after binding of the inhibitor to the other RAF monomer within a dimer [187,188]. New, second generation inhibitors have been developed in the last decade with the ability to brake the paradoxical ERK activation, and many of them are currently being analyzed in clinical trials [189]. In addition to RAF inhibitors, MEK and ERK inhibitors have also been developed, but these inhibitors have narrow therapeutic dose margin due to their effect on normal cells. Despite this, two MEK inhibitors (cobimetinib and trametinib) are currently being used for treatment of tumors harboring BRAF mutations [190].

### 6.3. Direct RAS Inhibitors

The initial notion that RAS was undruggable began to change with the discovery of pockets on the RAS surface that could be used to design new specific inhibitors capable of binding directly to the RAS oncoproteins. Based on structural and crystallographic studies showing that HRAS proteins cycle between two structural states (called state 1 and state 2) and that RAS proteins are unable to bind to effectors while in state 1, the existence of a groove in the surface of HRAS in state 1 was predicted that would be large enough to bind small molecule inhibitors potentially capable of blocking RAS-effector interaction [191]. This seminal report started a race to find and develop new small molecules that could block RAS interaction with either its effectors, guanine nucleotides, or its GEF activators. In this regard, many reports have been published describing the isolation of small molecule RAS inhibitors, either blocking RAS/effector interactions [192] or inhibiting the interaction with SOS proteins (rev. in [55]). Happily, some of these inhibitors have already overcome most barriers in drug development and are being tested in the clinic. For example, Sulindac and Rigosertib (drugs that inhibit RAS/RAF interaction) have reached Phase 3 clinical trials for several human malignancies, and BI-1701963 is the first inhibitor of RAS/SOS interaction to reach clinical trials (for more information see https://www.clinicaltrials.gov, accessed on 5 April 2021). 

One of the most relevant developments in the race towards blocking RAS in cancer came from the discovery of a small-molecule covalent inhibitor of KRAS^G12C^ that strongly and irreversibly bound to the mutated cysteine and prevented guanine nucleotide exchange, RAS activation and cancer cell growth [193]. This initial drug had limited inhibitory capacity but opened the gate for the development of new, more potent inhibitors in the following years that are able to bind to both RAS.GDP and RAS.GTP complexes, even independently of the specific oncogenic mutation carried, in different biological and tumoral contexts (rev. in [194]). At the beginning of 2021 there were 15 clinical trials listed in *Clinical Trials.gov* dedicated to analyzing the effectiveness and toxicity of KRAS^G12C^ inhibitors in solid tumors; especially lung cancers, where this mutation is frequent. Two of these inhibitors (AMG 510 [195] and MRTX849 [196]) have reached phase 3 clinical trials but, unfortunately, resistance mechanisms have already been described and further work will be needed to overcome them in the future [196].

### 6.4. Future Perspectives on RAS Therapy

RAS research is entering its fifth decade, behind more than forty years of discoveries that have revealed a vast amount of information on these small GTPases. From the discovery of the viral oncogenes, and the isolation of their cellular counterparts, to the latest work on RAS inhibition and signaling, the knowledge on these proteins has allowed to design new strategies to target their role in cancer. Thus, new avenues to inhibit RAS translocation to the plasma membrane, to block downstream signaling pathways, or to directly inhibit their activity through small inhibitor binding, have come out in the last ten years, and covalent KRAS^G12C^ inhibitors might be the first drugs targeting RAS directly that may reach FDA approval for clinical use.

Despite these recent advances, there is still a long way to go to effectively block RAS signaling in cancer. KRASG12C mutations comprise only a small percentage of RAS alterations in cancer and new drugs specifically targeting other RAS mutations are needed. Furthermore, resistance to drugs targeting RAS will likely limit their efficacy in the clinic [197], and effective treatments may probably require combinatorial therapies. Another concern regarding the development of effective RAS inhibitors is toxicity. Recent research had shown that complete abrogation of most members of RAS signaling pathways in mice leads to death; predicting a high toxicity for inhibitors that effectively block them [198], a problem that can be extended to most chemotherapeutic tools.

Another relevant consideration for therapy of RAS-driven cancer is the long-known ability of RAS oncogenes to cause accumulation of genomic/chromosomal instability that critically contributes to progression through the evolutionary phases of tumorigenesis [199,200]. This suggests that future therapeutic approaches against RAS-driven cancers should not only focus on searching for new biochemical inhibitors directly binding to RAS surface pockets, but also for wider approaches capable of targeting various other mechanistic aspects and hallmarks of RAS-dependent cancer evolution [201], as well as reducing systemic toxicity by specifically releasing the chemotherapeutic drugs to the tumors [202].

## Data Availability

Not applicable.
